# Supplementary material for: Sequence Variant Analysis of the APOCII Locus among an Arab Cohort
Source: Int J Mol Sci. 2023 Nov 14;24(22):16293. doi: 10.3390/ijms242216293 (PMC10671382; doi:10.3390/ijms242216293)
Supplement: Supplementary file 1 [file ijms-24-16293-s001.zip › ijms-2705019-supplementary.pdf]

Supplementary Table S1. The genotype distribution for the identified 45 SNPs at the full APOCII gene locus among the four studied groups (n = 200).

	SNP ID	Kuwaiti Arab HTG	Kuwaiti Non-Arab T2DM	Kuwait Arab T2DM	Controls of Arab ethnicity
1	rs10425530	G/G = 49	G/G = 50	G/G = 50	G/G = 49
		G/A = 1	G/A = 0	G/A = 0	G/A = 1
		A/A = 0	A/A = 0	A/A = 0	A/A = 0
		**0.943**	NA	**NA**	**0.943**
2	rs111782345	G/G = 49	G/G = 50	G/G = 49	G/G = 49
		G/A = 1	G/A = 0	G/A = 1	G/A = 1
		A/A = 0	A/A = 0	A/A = 0	A/A = 0
		**0.943**	**NA**	**0.943**	**0.943**
3	rs112698600	C/C = 49	C/C = 50	C/C = 49	C/C = 49
		C/T = 1	C/T = 0	C/T = 1	C/T = 1
		T/T = 0	T/T = 0	T/T = 0	T/T = 0
		**0.943**	**NA**	**0.943**	**0.943**
4	rs111356234	G/G = 48	G/G = 50	G/G = 50	G/G = 50
		G/A = 2	G/A = 0	G/A = 0	G/A = 0
		A/A = 0	A/A = 0	A/A = 0	A/A = 0
		**0.885**	**NA**	**NA**	**NA**
5	rs12721063	G/G = 50	G/G = 50	G/G = 49	G/G = 49
		G/A = 0	G/A = 0	G/A = 1	G/A = 1
		A/A = 0	A/A = 0	A/A = 0	A/A = 0
		**NA**	**NA**	**0.943**	**0.943**
6	rs2288912	C/C = 10	C/C = 3	C/C = 10	C/C = 12
		C/G = 24	C/G = 37	C/G = 25	C/G = 26
		G/G = 16	G/G = 10	G/G = 15	G/G = 12
		**0.854**	0.0003	**0.943**	**0.777**
7	KUA_ApoCII_1	G/G = 50	G/G = 49	G/G = 50	G/G = 50
		G/A = 0	G/A = 1	G/A = 0	G/A = 0
		A/A = 0	A/A = 0	A/A = 0	A/A = 0
		**NA**	**0.943**	**NA**	**NA**
8	rs12709886	C/C = 49	C/C = 49	C/C = 48	C/C = 50
		C/T = 1	C/T = 1	C/T = 2	C/T = 0
		T/T = 0	T/T = 0	T/T = 0	T/T = 0
		**0.943**	**0.943**	**0.885**	**NA**
9	rs9304644	C/C = 14	C/C = 11	C/C = 17	C/C = 15

		C/T = 21	C/T = 29	C/T = 21	C/T = 23
		T/T = 15	T/T = 10	T/T = 12	T/T = 12
		**0.259**	**0.257**	**0.0230**	**0.588**
10	rs527585098	G/G = 50	G/G = 50	G/G = 49	G/G = 50
		G/A = 0	G/A = 0	G/A = 1	G/A = 0
		A/A = 0	A/A = 0	A/A = 0	A/A = 0
		**NA**	**NA**	**0.943**	**NA**
11	rs12721076	T/T = 33	T/T = 31	T/T = 38	T/T = 41
		T/C = 17	T/C = 16	T/C = 11	T/C = 9
		C/C = 0	C/C = 3	C/C = 1	C/C = 0
		**0.148**	**0.633**	**0.846**	**0.484**
12	KUA_ApoCII_2	T/T = 50	T/T = 50	T/T = 49	T/T = 50
		T/C = 0	T/C = 0	T/C = 1	T/C = 0
		C/C = 0	C/C = 0	C/C = 0	C/C = 0
		**NA**	**NA**	**0.943**	**NA**
13	rs9304645	G/G = 27	G/G = 27	G/G = 28	G/G = 29
		G/A = 21	G/A = 21	G/A = 20	G/A = 20
		A/A = 2	A/A = 2	A/A = 2	A/A = 1
		**0.396**	**0.396**	**0.495**	**0.242**
14	rs9304646	T/T = 14	T/T = 11	T/T = 16	T/T = 17
		T/C = 22	T/C = 28	T/C = 22	T/C = 22
		C/C = 14	C/C = 11	C/C = 12	C/C = 11
		**0.396**	**0.396**	**0.419**	**0.449**
15	rs920667500	T/T = 49	T/T = 50	T/T = 50	T/T = 50
		T/C = 1	T/C = 0	T/C = 0	T/C = 0
		C/C = 0	C/C = 0	C/C = 0	C/C = 0
		**0.943**	**NA**	**NA**	**NA**
16	rs534276172	G/G = 49	G/G = 50	G/G = 50	G/G = 50
		G/T = 1	G/T = 0	G/T = 0	G/T = 0
		T/T = 0	T/T = 0	T/T = 0	T/T = 0
		**0.943**	**NA**	**NA**	**NA**
17	rs11879392	C/C = 34	C/C = 31	C/C = 40	C/C = 41
		C/G = 16	C/G = 17	C/G = 9	C/G = 9
		G/G = 0	G/G = 2	G/G = 1	G/G = 0
		**0.178**	**0.861**	**0.568**	**0.484**
18	rs10419086	A/A = 44	A/A = 45	A/A = 41	A/A = 43
		A/G = 6	A/G = 5	A/G = 9	A/G = 6
		G/G = 0	G/G = 0	G/G = 0	G/G = 1

		**0.652**	**0.709**	**0.484**	**0.191**
19	rs4803774	A/A = 14	A/A = 12	A/A = 15	A/A = 15
		A/G = 21	A/G = 28	A/G = 24	A/G = 23
		G/G = 15	G/G = 10	G/G = 11	G/G = 12
		**0.259**	**0.389**	**0.811**	**0.588**
20	rs4803775	T/T = 10	T/T = 8	T/T = 11	T/T = 12
		T/C = 23	T/C = 31	T/C = 24	T/C = 21
		C/C = 17	C/C = 11	C/C = 15	C/C = 17
		**0.663**	**0.083**	**0.811**	**0.284**
21	rs772477913	T/T = 50	T/T = 49	T/T = 50	T/T = 50
		T/C = 0	T/C = 1	T/C = 0	T/C = 0
		C/C = 0	C/C = 0	C/C = 0	C/C = 0
		**NA**	**0.943**	**NA**	**NA**
22	rs151176577	G/G = 50	G/G = 50	G/G = 49	G/G = 50
		G/A = 0	G/A = 0	G/A = 1	G/A = 0
		A/A = 0	A/A = 0	A/A = 0	A/A = 0
		**NA**	**NA**	**0.943**	**NA**
23	rs12721058	C/C = 50	C/C = 50	C/C = 50	C/C = 49
		C/G = 0	C/G = 0	C/G = 0	C/G = 1
		G/G = 0	G/G = 0	G/G = 0	G/G = 0
		**NA**	**NA**	**NA**	**0.943**
24	KUA_ApoCII_3	A/A = 49	A/A = 50	A/A = 50	A/A = 50
		A/C = 1	A/C = 0	A/C = 0	A/C = 0
		C/C = 0	C/C = 0	C/C = 0	C/C = 0
		**0.943**	**NA**	**NA**	**NA**
25	rs10420434	G/G = 44	G/G = 45	G/G = 43	G/G = 43
		G/A = 6	G/A = 5	G/A = 7	G/A = 6
		A/A = 0	A/A = 0	A/A = 0	A/A = 1
		**0.652**	**0.709**	**0.594**	**0.191**
26	KUA_ApoCII_4	C/C = 49	C/C = 50	C/C = 50	C/C = 50
		C/T = 1	C/T = 0	C/T = 0	C/T = 0
		T/T = 0	T/T = 0	T/T = 0	T/T = 0
		**0.943**	**NA**	**NA**	**NA**
27	rs555315266	G/G = 49	G/G = 50	G/G = 50	G/G = 50
		G/A = 1	G/A = 0	G/A = 0	G/A = 0
		A/A = 0	A/A = 0	A/A = 0	A/A = 0
		**0.943**	**NA**	**NA**	**NA**
28	rs7256684	A/A = 14	A/A = 12	A/A = 16	A/A = 15

		A/G = 21	A/G = 28	A/G = 22	A/G = 22
		G/G = 15	G/G = 10	G/G = 12	G/G = 13
		**0.259**	**0.389**	**0.419**	**0.402**
29	rs12721060	T/T = 49	T/T = 50	T/T = 49	T/T = 49
		T/G = 1	T/G = 0	T/G = 1	T/G = 1
		G/G = 0	G/G = 0	G/G = 0	G/G = 0
		**0.943**	**NA**	**0.943**	**0.943**
30	rs1019828365	C/C = 50	C/C = 50	C/C = 48	C/C = 50
		C/T = 0	C/T = 0	C/T = 2	C/T = 0
		T/T = 0	T/T = 0	T/T = 0	T/T = 0
		**NA**	**NA**	**0.885**	**NA**
31	rs5120	T/T = 10	T/T = 7	T/T = 12	T/T = 12
		T/A = 23	T/A = 32	T/A = 23	T/A = 20
		A/A = 17	A/A = 11	A/A = 15	A/A = 18
		**0.663**	**0.0410**	**0.588**	**0.183**
32	rs7257095	C/C = 47	C/C = 47	C/C = 47	C/C = 45
		C/G = 3	C/G = 3	C/G = 3	C/G = 5
		G/G = 0	G/G = 0	G/G = 0	G/G = 0
		**0.827**	**0.827**	**0.827**	**0.709**
33	rs10422603	T/T = 28	T/T = 26	T/T = 30	T/T = 29
		T/G = 20	T/G = 21	T/G = 17	T/G = 20
		G/G = 2	G/G = 3	G/G = 3	G/G = 1
		**0.495**	**0.644**	**0.777**	**0.242**
34	rs373211202	C/C = 49	C/C = 50	C/C = 50	C/C = 50
		C/A = 1	C/A = 0	C/A = 0	C/A = 0
		A/A = 0	A/A = 0	A/A = 0	A/A = 0
		**0.943**	**NA**	**NA**	**NA**
35	rs112265403	C/C = 50	C/C = 50	C/C = 50	C/C = 49
		C/T = 0	C/T = 0	C/T = 0	C/T = 1
		T/T = 0	T/T = 0	T/T = 0	T/T = 0
		**NA**	**NA**	**NA**	**0.943**
36	rs3745152	G/G = 36	G/G = 40	G/G = 39	G/G = 37
		G/T = 0	G/T = 0	G/T = 0	G/T = 0
		T/T = 14	T/T = 10	T/T = 11	T/T = 13
		**0.000**	**0.000**	**0.000**	**0.000**
37	rs4803776	T/T = 31	T/T = 34	T/T = 34	T/T = 34
		T/C = 4	T/C = 6	T/C = 5	T/C = 2
		C/C = 15	C/C = 10	C/C = 11	C/C = 14

		0.000	0.000	0.000	0.000
38	rs180809422	A/A = 49	A/A = 50	A/A = 49	A/A = 49
		A/C = 1	A/C = 0	A/C = 1	A/C = 1
		C/C = 0	C/C = 0	C/C = 0	C/C = 0
		**0.943**	**NA**	**0.943**	**0.943**
39	KUA_ApoCII_5	G/G = 50	G/G = 50	G/G = 50	G/G = 49
		G/C = 0	G/C = 0	G/C = 0	G/C = 1
		C/C = 0	C/C = 0	C/C = 0	C/C = 0
		**NA**	**NA**	**NA**	**0.943**
40	rs1130742	C/C = 46	C/C = 49	C/C = 47	C/C = 48
		C/T = 0	C/T = 0	C/T = 0	C/T = 0
		T/T = 4	T/T = 1	T/T = 3	T/T = 2
		0.000	0.000	0.000	0.000
41	rs10421404	G/G = 28	G/G = 24	G/G = 28	G/G = 29
		G/A = 14	G/A = 17	G/A = 13	G/A = 11
		A/A = 8	A/A = 9	A/A = 9	A/A = 10
		0.018	**0.073**	0.006	0.000
42	rs7257468	C/C = 23	C/C = 23	C/C = 24	C/C = 26
		C/T = 14	C/T = 17	C/T = 15	C/T = 11
		T/T = 13	T/T = 10	T/T = 11	T/T = 13
		0.003	0.056	0.011	0.000
43	rs7258345	T/T = 23	T/T = 23	T/T = 24	T/T = 26
		T/G = 14	T/G = 17	T/G = 15	T/G = 11
		G/G = 13	G/G = 10	G/G = 11	G/G = 13
		0.003	0.056	0.011	0.000
44	rs7257476	C/C = 23	C/C = 23	C/C = 24	C/C = 26
		C/T = 14	C/T = 17	C/T = 15	C/T = 11
		T/T = 13	T/T = 10	T/T = 11	T/T = 13
		0.003	0.056	0.011	0.000
45	rs12709889	G/G = 31	G/G = 32	G/G = 33	G/G = 29
		G/A = 15	G/A = 17	G/A = 14	G/A = 19
		A/A = 4	A/A = 1	A/A = 3	A/A = 2
		**0.279**	**0.459**	**0.377**	**0.607**

Supplementary Table s2. Minor allele frequency distribution among different populations and their reported consequence.

SNP ID	Global	AFR	EAS	SAS	AMR	Kuwait n=200	NHW n=95*	AB n=95*	Associated metabolic disorders	Reference
**Common Variants: MAF≥0.05 in Global**										
rs2288912	0.660	0.763	0.675	0.640	0.730	0.560	0.484	0.232	AD, GERD, CAD, High LDL, Increased C-Reactive protein levels, High VLSFAs	Adewuyi, E.O et al., 2022 & Lemaitre, R.N et al., 2015 [36,37].
rs9304644	0.558	0.572	0.592	0.590	0.590	0.480	0.457	0.358	Family history of AD decreased Omega 3	Marioni, R.E et al., 2018 & Richardson, T.G et al., 2022 [38,39].
rs12721076	0.116	0.031	0.058	0.250	0.160	0.153	0.154	0.032		
rs9304645	0.223	0.330	0.079	0.310	0.210	0.237	0.188	0.394		Rodrigues, J.C.G et al., 2021 [40].
rs9304646	0.574	0.621	0.595	0.600	0.600	0.475	0.457	0.288		
rs113329239	0.565	0.597	0.593	0.600	0.590	0.500	0.000	0.000		
rs11879392	0.117	0.031	0.059	0.260	0.160	0.143	0.153	0.027		
rs10419086	0.097	0.188	0.061	0.040	0.140	0.070	0.063	0.142		
rs4803774	0.575	0.622	0.598	0.600	0.600	0.480	0.453	0.289	Family history of AD	Schwartzentruber, J et al., 2021 [41].
rs4803775	0.670	0.803	0.659	0.640	0.730	0.548	0.484	0.158		
rs10420434	0.096	0.182	0.067	0.040	0.130	0.065	0.063	0.126	Increased C-Reactive	Koskeridi F et al., 2022 [42].

									protein levels	
rs7256684	0.574	0.623	0.590	0.600	0.600	0.483	0.453	0.287		
rs5120	0.670	0.803	0.656	0.640	0.730	0.55	0.484	0.158		
rs7257095	0.052	0.135	0.000	0.060	0.020	0.035	0.000	0.163		
rs10422603	0.214	0.285	0.083	0.310	0.210	0.24	0.189	0.306		
rs10622462	0.568	0.607	0.587	0.600	0.600	0.475	0.000	0.000		
rs3745152	0.568	0.607	0.587	0.600	0.600	0.240	0.489	0.259		
rs4803776	0.669	0.802	0.656	0.640	0.730	0.293	0.483	0.179		
rs150448996	0.329	0.247	0.503	0.260	0.380	0.225	0.261	0.272		
rs1130742	0.329	0.247	0.503	0.260	0.380	0.050*	0.295	0.279	Decreased blood protein levels	
rs10421404	0.211	0.259	0.083	0.330	0.200	0.318	0.189	0.305	Increased LDL	Hoffmann, T.J et al., 2018 [43].
rs78403558	0.124	0.055	0.057	0.260	0.170	0.185	0.153	0.508		
rs7257468	0.569	0.604	0.588	0.600	0.600	0.378	0.458	0.284		
rs7258345	0.569	0.606	0.588	0.600	0.600	0.378	0.457	0.285		
rs7257476	0.569	0.606	0.588	0.600	0.600	0.378	0.458	0.295		
rs12709889	0.323	0.231	0.502	0.260	0.380	0.213	0.268	0.250		
**Common Variants: MAF 0.01-0.05 in Global**										
rs10425530	0.033	0.116	0.003	0.000	0.010	0.005	0.000	0.101		
**Common Variants: MAF≤0.01 in Global**										
rs371259300	0.008	0.028	0.000	0.000	0.000	0.500	0.000	0.000		
rs111782345	0.005	0.015	0.002	0.000	0.000	0.008	0.000	0.005		
rs112698600	0.004	0.014	0.000	0.000	0.000	0.008	0.000	0.011		

rs111356234	0.007	0.026	0.000	0.000	0.000	0.005	0.000	0.053		
rs12721063	0.002	0.002	0.000	0.000	0.000	0.005	0.021	0.000	Increased C-Reactive protein levels	Sinnott-Armstrong, N et al., 2021 [44].
rs12709886	0.007	0.000	0.000	0.010	0.010	0.01	0.037	0.000	Increased C-Reactive protein levels	Koskeridi F et al., 2022 [42].
rs527585098	0.000	0.001	0.000	0.000	0.000	0.003	0.000	0.000		
rs920667500	0.000	0.000	0.000	0.000	0.000	0.003	0.000	0.000		
rs534276172	0.000	0.000	0.000	0.000	0.000	0.003	0.000	0.000		
rs151176577	0.004	0.012	0.000	0.000	0.000	0.003	0.000	0.011		
rs12721058	0.003	0.011	0.000	0.000	0.000	0.003	0.000	0.000		
rs555315266	0.000	0.001	0.001	0.000	0.000	0.003	0.000	0.000		
rs12721060	0.005	0.017	0.000	0.000	0.000	0.008	0.000	0.011		
rs1019828365	0.003	0.000	0.000	0.000	0.000	0.005	0.000	0.000		
rs569591876	0.001	0.002	0.000	0.000	0.000	0.003	0.000	0.000		
rs373211202	0.003	0.000	0.000	0.000	0.000	0.003	0.000	0.000		
rs112265403	0.004	0.017	0.000	0.000	0.000	0.003	0.000	0.000		
rs180809422	0.000	0.014	0.000	0.000	0.000	0.008	0.00	0.011		

AFR: African, EAS: East Asian, SAS: South Asian, AMR: American, NHW: Non-Hispanic Whites, AB: African Blacks, AD: Alzheimer disease, GERD: gastroesophageal reflux disease, VLCFA: very-long-chain saturated fatty acids, * SNP deviated from Hardy-Weinberg equilibrium, † novel SNP and * in Pirim et al 2019 [1].

Reference

1. Pirim, D.; Radwan, Z.H.; Wang, X.; Niemsiri, V.; Hokanson, J.E.; Hamman, R.F.; Feingold, E.; Bunker, C.H.; Demirci, F.Y.; Kamboh, M.I. Apolipoprotein E-C1-C4-C2 gene cluster region and inter-individual variation in plasma lipoprotein levels: A comprehensive genetic association study in two ethnic groups. *PLoS ONE* **2019**, *14*, e0214060.
